# Atherogenic Factors and Their Epigenetic Relationships

**DOI:** 10.1155/2010/437809

**Published:** 2010-09-16

**Authors:** Ana Z. Fernandez, Andrew L. Siebel, Assam El-Osta

**Affiliations:** ^1^Hemostasia and Vascular Genetics Laboratory, Biophysics and Biochemistry Center, Venezuelan Institute for Scientific Research IVIC, Carretera Panamericana km11, P.O. 26973, Caracas 1020, Venezuela; ^2^Epigenetics in Human Health and Disease Laboratory, Baker IDI Heart and Diabetes Institute, Melbourne, VIC 3004, Australia

## Abstract

Hypercholesterolemia, homocysteine, oxidative stress, and hyperglycemia have been recognized as the major risk factors for atherogenesis. Their impact on the physiology and biochemistry of vascular cells has been widely demonstrated for the last century. However, the recent discovery of the role of epigenetics in human disease has opened up a new field in the study of atherogenic factors. Thus, epigenetic tags in endothelial, smooth muscle, and immune cells seem to be differentially affected by similar atherogenic stimuli. This paper summarizes some recent works on expression of histone-modifying enzymes and DNA methylation directly linked to the presence of risk factors that could lead to the development or prevention of the atherosclerotic process.

## 1. Introduction

Cardiovascular-related diseases are the most serious threat to human health that authorities will have to manage worldwide. All of these pathological outcomes, like stroke, thrombosis, or infarction, are pathological events resulting from the long-lasting and silent process, known as atherosclerosis [1]. Atherosclerosis is considered to be a multifactorial pathology where preferential zones within the arteries, such as branches and curvatures, are prone to differential expression of genes with a proatherosclerotic profile, serving as potential substratum for lesions when risk factors are introduced [2, 3]. In this sense epigenetics, defined as the structural adaptation of chromosomal regions so as to register, signal, or perpetuate altered gene activity states [4] might play a significant role in the pathogenicity of cardiovascular risk factors, which could explain the specificity in the establishment and further development of the atherosclerotic lesion.

## 2. Atherosclerosis Overview

Atherosclerosis is a progressive disease characterized by the accumulation of lipids and fibrous elements in susceptible zones in the large and medium arteries [5]. Normal arteries are characterized by one internal or luminal surface of nonadherent endothelial cells (ECs) over a layer of extracellular matrix (ECM), mainly formed by collagen and proteoglycans, followed by a media layer of smooth muscle cells (SMC) and an external adventitia layer. Diverse stimuli, such as hypercholesterolemia, smoking, or hypertension, lead to a pathological activation of EC, attracting blood monocytes into the intima layer, where they differentiate into macrophages in order to remove cholesterol accumulated in this layer. Monocyte chemoattractant protein-1 (MCP-1) appears responsible for the direct migration of monocytes into the intima at sites of lesion formation, and macrophage colony-stimulating factor (M-CSF) contributes to the differentiation of monocytes into macrophages [6], while vascular cell adhesion molecule-1 (VCAM-1) expressed earlier on endothelial surface binds monocytes and T lymphocytes [7]. The by-products of cholesterol-loaded macrophages attract other immune cells and promote the phenotypic switching of SMC, transforming them from a “contractile” to a “synthetic” phenotype [8]. Early atherosclerotic lesions can be found even in the fetal aorta, enhanced greatly by maternal hypercholesterolemia [9].

## 3. Structural Epigenetic Modifications on the Chromatin

**Figure 1:**
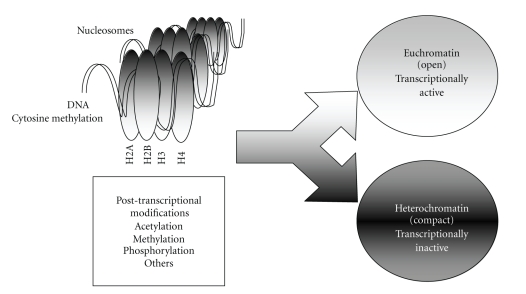
The basic chromatin unit or nucleosome consists of a protein octamer containing two molecules of each histone (H2A, H2B, H3 and H4), around which DNA is wrapped. The arrangement of nucleosomes can be altered by posttranscriptional modification of histones, that could be associated to heterochromatin, highly compacted silenced chromatin, or euchromatin, transcriptionally active open chromatin.

In eukaryotes, the protection and packaging of the genetic material are largely performed by the histone proteins, which also offer a scaffold for regulating processes such as transcription, replication, and repair of DNA [10]. Chromatin, the DNA-nucleosome polymer, is a dynamic molecule existing in many configurations between heterochromatin, highly compacted and silenced chromatin, and euchromatin, transcriptionally active open chromatin [11]. The basic chromatin unit or nucleosome consists of a protein octamer containing two molecules of each canonical histone (H2A, H2B, H3, and H4), around which is wrapped 147 base pairs of DNA (Figure 1). Nucleosomes may be irregularly packed and fold into higher-order structures that occur in diverse regions of the genome during cell-fate specification or in distinct stages of the cell cycle. The arrangement of nucleosomes can be altered by covalent modification of histones, which can include acetylation, methylation, phosphorylation, ubiquitination, and sumoylation. Posttranslational modifications of these proteins are facilitated by different enzymes, whose activities are regulated by other specific modulators. Although the enzymes involved in histone modifications are sequence-specific surrounding the target amino acid residue, it is widely believed that they may possess substrates outside the histone molecules [12]. 

**Figure 2:**
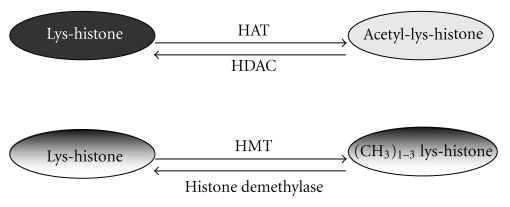
Main posttranslational modifications occurring on Histone 3 and 4 are the result of dynamic activity of different enzymes. HAT: histone acetyl transferase; HDAC: histone deacetylase; HMT: histone methyl transferase; (CH_3_)_1−3_: Lys can be mono-, di, or trimethylated

Acetylated-lysines in histone 3 (H3) and histone 4 (H4) associated with active transcription are the result of the interplay between histone acetylases and deacetylases (Figure 2). Histone acetyltransferase (HAT; EC 2.3.1.48) catalyzes the transfer of an acetyl group from acetyl-CoA to specific lysines in histone. Transcription coactivators, like p300/Creb-Binding Protein (PCAF), display acetyltransferase activity [13]. Histone deacetylase (HDAC; EC 3.5.1.98) comprises a range of isoenzymes responsible for catalyzing the removal of acetyl groups from lysine residues of histones. They are classified as class I, II, or III according to homologous sequence domains. HDAC activity is inhibited by butyrate, trichostatin A (TSA), and suberoylanilide hydroxamic acid (SAHA), with the list of HDAC inhibitors growing due to the increasing interest in their effects in a clinical setting as anticancer drugs [14]. TSA is the most potent HDAC inhibitor known to date and is frequently used in studies on the role of histone acetylation in gene expression. Although it has been approved the use of some HDAC inhibitors in cancer therapy, the major concern is their lack of specificity [15]. 

Histone methylation is also a major dynamic covalent epigenetic modification (Figure 2). The lysine residue modified can be mono-, di-, or trimethylated and, depending on the position in the histone chain, methylated lysines are associated with transcriptional activation or gene suppression. Histone-lysine N-methyltransferase (HMTase, EC. 2.1.1.43) catalyzes the transfer of a methyl group from S-adenosyl-L-methionine to a lysine residue either on H3 or H4, while histone demethylases eliminate methyl groups [16]. 

On the other hand, DNA itself can be modified covalently by methylation at the cytosine residue of CpG dinucleotides, which commonly leads to suppression of gene expression when occurring in a regulatory region [11]. DNA methylation at the promoter region can control gene transcription by recruiting methylcytosine-binding proteins (MBPs) which recognize methylated DNA sequences. Thus, DNA methyltransferases (DNMT, EC 2.1.1.37) catalyze the transfer of methyl groups to DNA from S-adenosylmethionine (SAM). DNMT can be grouped into four distinct families based on sequence homology within their C-terminal catalytic domains: DNMT1, DNMT2, and DNMT3 families and the chromomethylase family, unique to the plant kingdom [17]. In mammals, DNMT1 is the maintenance methyltransferase, which preferentially methylates hemimethylated double-stranded DNA, whereas DNMT3 is known as the de novo methyltransferase, which methylates unmethylated or hemimethylated double-stranded DNA. The mammalian genome encodes two functional cytosine methyltransferases of the DNMT3 family, DNMT3A and DNMT3B, and a third homologue, DNMT3L, which lacks cytosine methyltransferase activity and functions as a regulatory factor in germ cells. DNMT3A and DNMT3B are expressed in a range of adult tissues but at lower levels than DNMT1 [17].

## 4. Main Proatherogenic Risk Factors and Epigenetic-Associated Mechanisms

Atherosclerosis is a complex disease caused by both genetic and environmental factors. More than one hundred atherosclerosis-related genes have been discovered through different approaches such as genetically modified organisms (apoE or LDL receptor knockout mice) or nutritional overload (fat-feeding). Many of the genes encode for coagulation factors, apolipoproteins and lipoprotein receptors, the blood pressure axis, ECM proteases, and inflammatory modulators, among others [18–20]. However, the establishment of a proatherogenic profile is strongly correlated with the presence of one or more risk factors, including cholesterol, homocysteine, oxidative stress, and diabetes. The molecular effects of these risk factors on EC, SMC, and monocyte/macrophages have been widely studied; however the study of the effects on histone or DNA modifications is still growing. 

### 4.1. Cholesterol

The causal relationship between hypercholesterolemia and atherosclerosis was identified over 100 years ago, but evidence was not clear enough to be considered by physicians until three or four decades ago (reviewed in [21]). The discovery of the plasma cholesterol-carriers (i.e., lipoproteins) and the association of different kinds of lipoproteins with cardiovascular disease development was a major milestone in the atherosclerosis research field. Thus, both elevated low-density lipoprotein (LDL) and reduced high-density lipoprotein (HDL) lead to an enhanced risk of coronary heart disease. A comparative evaluation of the two risk factors in the Atherosclerosis Risk in communities (ARIC) study supports the idea that the degree of coronary risk is similarly affected by high LDL-C and low HDL-C [22].

In an *in vivo* model, Alkemade et al. [23] demonstrated that both *in utero* programming and diet-induced hypercholesterolemia affected histone methylation modifications and expression of accompanying lysine methyltransferases in vascular EC and SMC, in 20-week old female apoE^+/−^ mouse offspring from apoE^−/−^ and wild-type mothers.Furthermore, these epigenetic modifications were different in each cell type, which suggest cell-specific epigenetic effects in response to a similar stimuli [23].

### 4.2. Homocysteine

Homocysteine is a nonessential sulfur-containing amino acid involved in methionine metabolism that has been gaining greater interest during the last four decades due to its association with cardiovascular diseases [24, 25]. The pathogenetic mechanism for hyperhomocysteinemia is complex, since it requires the coexistence of genetic defects of the enzymes in the homocysteine-methionine metabolic pathway and a disturbed nutritional state regarding folic acid, vitamin B12, and vitamin B6, which act as cofactors of the enzymes regulating the metabolism of homocysteine [26–28].

Hyperhomocysteinemia has been associated with a global reduction in genomic DNA methylation, as well as a downregulation of the intracellular SAM/S-adenosylhomocysteine (SAH) ratio, which is a marker of the methylation status of the cell [29]. In addition, an increase in DNMT3A and DNMT3B has been reported in SMC as a compensatory mechanism, since SAH inhibits SAM-dependent methyltransferases. DNA hypomethylation has been found on the cyclin A promoter of EC in the presence of 50 uM homocysteine, which leads to decreased binding of MeCP2 and increased binding of acetylated H3 and H4 to the cyclin A promoter in EC [30]. On the other hand, some critical promoter regions, such as PPAR*α* and PPAR*γ*, undergo cytosine methylation when human monocytes are incubated with higher levels of homocysteine [31].

### 4.3. Inflammation

Inflammation plays a central role in all phases of the atherosclerotic process [6]. T cells, monocytes-macrophages, and chemokines are the immune components involved in the formation of the atherosclerotic lesion. Thus, binding of monocytes to EC and SMC represents a key step in the pathogenesis of inflammation and atherosclerosis as well as antigen presentation by macrophages to T lymphocytes [6]. 

Most of the cytokines and chemokines are regulated by the nuclear factor kappa B transcription factor (NF*κ*B). NF*κ*B in the latent form exists in the cytoplasm of unstimulated cells comprising a transcriptionally active dimer bound to an inhibitor protein, IkB. The currently known subunit members of the NF*κ*B family in mammals are p50, p65 (RelA), c-Rel, p52, and RelB [32]. Both VCAM-1 and MCP-1 are under the transcriptional influence of NF*κ*B [33]. Chromatin modifications, such as histone lysine acetylation and arginine methylation, are key regulators of NF*κ*B activity. p65 protein is a key transcriptionally active component of NF*κ*B whose transactivation potential is enhanced by several coactivators, including PCAF and SRC1, which have histone acetyltransferase activity, and CARM1, which has arginine methyltransferase activity [34]. Monomethylation of H3K4 (active transcription marker) by the histone methyltransferase SET7 can regulate the expression of a subset of key NF*κ*B downstream proinflammatory genes, such as MCP-1 and IL-8, by interacting with NF*κ*B and modulating chromatin remodeling events at their promoters [34]. SET7 short hairpin (sh) RNA blocked the expression of proinflammatory genes in HEK-293 cells, as well as THP-1 monocytes. SET7 may therefore act as a novel coactivator of proinflammatory genes, opening the chromatin for enhanced transcription of a subset of NF*κ*B-dependent genes in diseases, such as atherosclerosis and diabetes [34, 35].

### 4.4. Oxidative Stress

The oxidation-reduction state of the cells is usually a balance between the generation of reactive oxygen species (ROS) and the specific molecular mechanisms to remove them. Once this equilibrium is in favor of higher levels of ROS, it is a condition known as oxidative stress. ROS include superoxide anion (O_2_^∗−^), hydrogen peroxide (H_2_O_2_), hydroxyl radical (OH*), hypochlorous acid (HOCl), nitric oxide (NO), and peroxynitrite (ONOO^−^). ROS can be generated in a variety of cell types, including SMC, EC, and mononuclear cells, and they are produced by various oxidase enzymes, including nicotinamide-adenine dinucleotide phosphate (NADPH) oxidase, xanthine oxidase, uncoupled endothelial NO synthase (eNOS), cyclooxygenase, glucose oxidase, and lipoxygenase, as well as via mitochondrial electron transport. Cells also have antioxidant mechanisms to protect themselves from the burden of ROS, such as superoxide dismutase (SOD), glutathione peroxidase (GPx), and catalases [36]. Enhanced oxidative stress has been shown to be an exacerbating factor in pathologies including inflammation, reperfusion injuries, and ageing [37]. Uncoupling of eNOS in the endothelium may lead to oxidative stress and endothelial dysfunction via numerous mechanisms [38]. Firstly, the enzymatic production of NO is diminished, allowing the radicals that it normally might react with to attack other cellular targets. Second, the enzyme begins to produce O_2_^∗−^, contributing to oxidative stress. Finally, it is likely that eNOS can become partially uncoupled, such that both O_2_^∗−^ and NO are produced simultaneously. Under these circumstances, eNOS may become a peroxynitrite generator, leading to a dramatic increase in oxidative stress [39]. 

Oxidation of LDL (oxLDL) produces minimally modified LDL, which has been involved in promotion of proatherosclerotic pathways [40]. Stimulation of HUVECs with oxLDL time dependently induced global, as well as specific IL-8 and MCP-1 promoter acetylation of H4 and phosphorylation/acetylation of H3 at Ser-10/Lys-14, probably via the LOX-1/ERK1/2 signaling pathway [40]. These effects are enhanced in the presence of HDAC inhibitors. Preincubation of cells with statins blocked oxLDL-related modification of H3 (Ser-10/Lys-14) as well as H4. These posttranscriptional modifications were accompanied by recruitment of NF*κ*B p65/RelA and RNA polymerase II to the promoters [40]. In addition, exposure of vascular SMCs to oxidative stress results in repression of the insulin-like growth factor 1 receptor (IGF1R), key receptor in the survival of many cells, as a result of epigenetic modifications involving the phosphorylation of p53 and its increased association with HDAC1 [41].

### 4.5. Diabetes Mellitus

Diabetes mellitus is characterized by accelerated atherosclerosis and higher risk for cardiovascular diseases. The hyperglycemia associated with diabetes can lead to modifications of macromolecules, for example, LDL, by forming advanced glycation end products (AGE), which can bind to surface receptors, such as RAGE (receptor for AGEs) [42]. These AGE-modified proteins can augment the production of proinflammatory cytokines and other inflammatory pathways [6]. The diabetic state also promotes oxidative stress and hyperglycemia, which increases the expression of MCP-1 in EC and SMC [43]. Moreover, it has been shown that high glucose levels promote foam cell formation, not only in macrophages but also in human cultured SMC [44].

 Inflammatory pathway activation in EC and SMC by high glucose levels has been related to epigenetic modifications [45, 46], and these modifications persisted after the return of the EC to physiological levels of glucose [45]. Thus, diabetes could activate inflammation pathways via epigenetic mechanisms.

## 5. Epigenetic Markers Linked to Atherosclerosis

**Figure 3:**
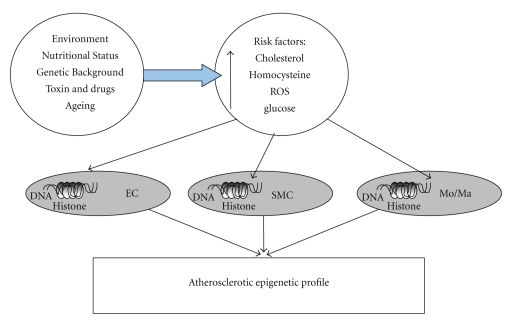
Factors such as nutritional status and genotype have been involved in the presence of risk factors, which not only activate biochemical pathways that lead to cellular dysfunction, but also can alter epigenetics tags on DNA and Histone. The sum of epigenetic modifications in the vascular cells will result in an atherosclerotic epigenetic profile. ROS: reactive oxygen species; EC: endothelial cell; SMC: smooth muscle cell; Mo/Ma: monocyte/macrophage.

The study of the epigenetic profile associated with proatherogenic gene activation or silencing in the vasculature may be complicated by the many distinct cell types involved in the process, as well as their genetic background, not to mention dietary and other environmental factors that could be involved (Figure 3). 

DNA methylation is an important epigenetic mechanism that selectively regulates gene expression and is associated with cardiovascular disease [47, 48]. Specific changes in DNA methylation pattern occur prior the appearance of vascular lesions in apoE^−/−^ mice, particularly in the aorta and circulating inflammatory cells [49].

The estrogen receptors *α* and *β* (ER*α* and ER*β*) are steroid hormone receptors that can act as transcription factors, both in vascular and nonvascular cells. ER*α* and ER*β* are important candidate genes to study epigenetic changes in atherosclerosis and vascular ageing as they have classical CpG islands in their promoter region, which can potentially modulate estrogenic cardiovascular protective effects [50]. Hypermethylation of ER*β* was observed in senescent vascular EC and SMC as well as in atherosclerotic lesions.

Histone acetylation seems to have a significant effect on atherogenesis. Choi et al. showed that TSA markedly exacerbated atherogenesis in hyperlipidemic mice, but without changing plasma lipid profiles. In addition, expression of mRNAs encoding CD36, SRA, TNF-alpha, and VCAM-1 was significantly elevated in aortic homogenates, although expression of MCP-1, IL-6, and IL-1 mRNA was decreased [51]. Furthermore, the expression of CD36 and the uptake of oxLDL were increased in a mouse monocyte-macrophage cell line incubated with 1 to 10 ng/ml of TSA. 

HDAC2 is implicated in the transcriptional inactivation of class II transactivator (CIITA), the transcriptional factor responsible for collagen promoter repression (COL1A2) in SMC and activation of Major Histocompatibility Complex ll (MHCII) promoter in macrophages and SMC, in response to IFN-*γ* [52]. HDAC2 interacts with and deacetylates CIITA, targeting it to proteasomal degradation, and antagonizing the transcriptional activity in macrophages and SMC. Therefore, HDAC2 may delay the atherogenic process by blocking T-cell activation and stabilizing the atherosclerotic plaque [52].

HDAC3 expression is upregulated in EC in areas close to branch openings where there is disturbed flow. HDAC3 knocked down in EC had a dramatic effect *in vitro* on EC morphology and survival, probably through interaction with Akt [51]. An *in vivo* mouse aortic isograft model was used to study the effect of HDAC3, and it confirmed a prominent role for the deacetylase enzyme in maintaining the integrity of the vessel [53]. Moreover, HDAC3 siRNA decreased the expression of VCAM-1 in endothelial cells and the adhesion of monocytes [54].

In steady laminar flow experiments, phosphorylation of HDAC5 was stimulated both in HUVEC and bovine aortic EC [55]. This phosphorylation was Ca2+/calmodulin dependent and also promoted the export of HDAC5 from the nucleus to the cytoplasm. The translocation of this HDAC released the transcriptional factor MEF2C, which could induce the expression of Kruppel like Factor 2 (KLF2), considered as an atheroprotective transcription factor [55, 56].

## 6. Perspectives

Given that epigenetics could represent the link between phenotype and genotype, it may be feasible to manipulate the epigenetic marks targeting the enzymes responsible for them to treat or prevent atherosclerosis, or at very least these biomarkers could be used as a measure of individual susceptibility to a given complication. There is evidence that each of the major risk factors linked to the atherosclerotic process has some degree of influence on the modulation of epigenetic tags that can be found on the promoter regions of transcription factors involved in the atherosclerotic process (i.e., NF*κ*B). It is the subsequent promotion or repression of transcription that controls expression of downstream genes in the associated pathway. Further studies should be directed both to the activation of protective genes in EC and/or SMC in sensitive zones of the vascular tree and the development of gene-specific silencing therapy through the regulation of cellular epigenetics.
